# Direct observations of X-rays produced by upward positive lightning

**DOI:** 10.1038/s41598-024-58520-x

**Published:** 2024-04-06

**Authors:** Toma Oregel-Chaumont, Antonio Šunjerga, Pasan Hettiarachchi, Vernon Cooray, Marcos Rubinstein, Farhad Rachidi

**Affiliations:** 1 Electromagnetic Compatibility Laboratory, Swiss Federal Institute of Technology (EPFL), 1015 Lausanne, VD Switzerland; 2 Faculty of Electrical Engineering, University of Split, 21000 Split, Croatia; 3 Department of Engineering Sciences, Uppsala University, 751 Uppsala, Sweden; 4 HEIG, University of Applied Sciences and Arts of Western Switzerland, 1401 Yverdon-les-Bains, VD Switzerland

**Keywords:** Lightning, X-rays, Leaders, Observations, Physics, Atmospheric dynamics, Atmospheric dynamics, Atmospheric dynamics, Electrical and electronic engineering

## Abstract

X-rays have been observed in natural downward cloud-to-ground lightning for over 20 years and in rocket-triggered lightning for slightly less. In both cases, this energetic radiation has been detected during the stepped and dart leader phases of downward negative flashes. More recently, X-rays have also been reported during the dart leader phase of *upward* negative flashes. In this study, we present the observations of four upward positive lightning flashes from the Säntis Tower (2.5 km ASL) in Switzerland. These consist of the simultaneous records of electric current passing through the tower, and electric field strength and X-ray flux 20 m from the tower base. One of the flashes was captured by a high-speed camera operating at 24,000 frames per second, stills from which are also presented. We detected X-rays during the initial phase of upward negative leader propagation, which can be associated with the leader-stepping process from electric field and current waveforms. To the best of our knowledge, this is the first time that such measurements are reported in the literature. The obtained time-synchronised data confirm that the X-ray emissions detected are associated with the initial steps of the upward negative leader. The frequency and energy of X-ray pulses appear to decrease as functions of time, with pulses disappearing altogether within the first millisecond of the leader initiation. X-ray emission also appears to be correlated with the maximum current-derivative and the electric field change of leader steps, consistent with cold electron runaway. These observations contribute to improving our understanding of upward lightning, which is a primary source of damage to tall structures such as wind turbines and telecommunications towers, as well as aircraft during takeoff and landing.

## Introduction

Lightning is known to produce electromagnetic radiation across a very wide spectrum, from radio waves to $\gamma$-rays^1^. Although they have been expected for some time, higher-energy emissions are a more recent discovery. In atmospheric air, X-rays and $\gamma$-rays are produced by the deceleration of relativistic electrons through “braking radiation” or *bremsstrahlung*. The first unambiguous observation of X-ray generation from lightning flashes was made by Moore et al.^2^, who recorded bursts of radiation with energies in excess of 1 MeV during the stepped-leader phase of three natural downward negative lightning flashes. Since then, X-ray emissions have been measured in both natural and artificially-triggered cloud-to-ground (CG) lightning via a series of experiments conducted at Camp Blanding, Florida^3–6^. Bursts of energetic radiation were detected during both the stepped-leader phase and dart leader–return stroke transition, with energies ranging from 100s of keV to 10s of MeV, this upper end being due to photon-burst energy pile-ups, rather than singular $\gamma$-rays, which are generally produced by terrestrial gamma-ray flashes (TGFs).

Measurements of X-ray emissions from natural upward lightning, however, were scanty until recently. Yoshida et al.^7^ observed increased counts associated with seven lightning flashes on their plastic and NaI scintillators designed for detecting high-energy electron and photon bursts, though the 1-ms sampling interval of their detectors did not permit precise identification of the emitting phase. Out of the seven, they reported results on two, an upward negative flash and an upward positive flash. Montanyà et al.^8^ made measurements of X-ray emissions from several upward lightning flashes from the mountaintop Eagle Nest tower located at 2537 m above sea-level (ASL) in the Pyrenées. They observed a 17 X-ray pulse burst (with an 806 keV maximum) during the stepped leader phase of a natural downward negative flash, but did not detect X-ray emissions during the 13 upward-initiated flashes reported. Hettiarachchi et al.^9^ were the first to directly measure X-ray emissions from upward-initiated lightning flashes: though either rare or very weak, they detected X-rays with energies up to 700 keV occurring both in bursts and as single events during the dart/dart-stepped leader phase of 3 natural upward negative flashes at Gaisberg Tower in Austria.

Herein we report, to the best of our knowledge, the first association of X-rays with the stepping of the upward negative leader in upward positive lightning flashes, as measured by the comprehensive Säntis lightning measurement system. The data consist of simultaneous records of lightning current and its derivative, near electric field (20 m), and high-speed camera (HSC) images. A summary of the data types analysed in this study and the aforementioned studies is presented in Table 1 [Interferometric (IFM) data is available but not analysed in this study.].

**Table 1:** Lightning X-ray measurement studies—a comparison.

Study	E-Field	Current	High-speed camera	Interferometer	Scintillators
Moore et al. 2001	Yes	Yes	No	No	1
Dwyer et al. 2003–2005	Yes	Yes	Yes	No	12
Yoshida et al. 2008	Yes	Yes	No	Yes	2
Saleh et al. 2009	Yes	Yes	No	No	45
Mallick et al. 2012	Yes	No	No	No	1
Montanyà et al. 2014	Yes	No	Yes	No	1
Hettiarachchi et al. 2018	Yes	Yes	No	No	2
This study	Yes	Yes	Yes	No	2

## Methods

**Figure 1:**
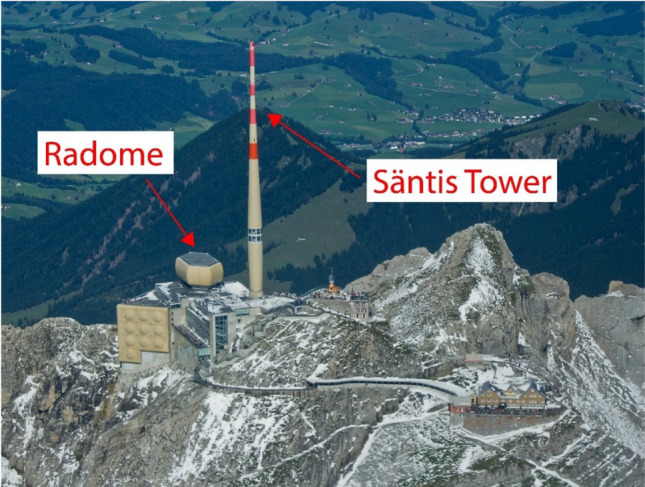
Photo of the Säntis peak, with arrows indicating the Radome, which houses the electric field probe and scintillators, and the Tower, where the current and current-derivative sensors are located. Image reproduced from Rachidi and Rubinstein^10^ (Fig. 2) with permission.

The Mt. Säntis Lightning Research Facility, shown in Fig. 1, is situated at 2502 m ASL in the Appenzell Alps of northeastern Switzerland, and experiences >100 direct lightning strikes per year to its 124 meter-tall tower, which is equipped with a Rogowski coil—$\dot{B}$ sensor pair at two different heights (24 and 82 meters above ground level), for measurement of the current and current derivative, respectively.[All four sensors operate with a sampling rate of 50 MHz, though the former has a lower frequency response (up to 2.4 MHz) than the latter (up to 25 MHz), meaning it may miss rapid changes, but capture slower ones that the latter cannot^11^. The nearby Radome houses an E-field sensor and two X-ray detectors (described below), which have a common sampling rate of 20 MHz. Our Mélopée fast E-field probe has a frequency range of 1 kHz to 150 MHz and is described in more detail in Šunjerga et al.^12^. Five kilometers away, atop Mt. Krönberg (1663 ASL), is a high-speed camera (HSC) operating at 24,000 fps, with an exposure time of 41 $\mu$s. Electric field measurements are also taken 15 km away by a flat-plate antenna with line-of-site in Herisau, Switzerland, though this data is not presented herein. Additionally, during the Summer of 2021, when the flashes discussed below occurred, a University of New Mexico interferometer (IFM) was installed in Schwägalp, at the base of Mt. Säntis. These interferometric results will be the subject of a separate paper.

One of our two NaI X-ray detectors belongs to Uppsala University and the other to the University of California—Santa Cruz (UCSC); the former records waveforms and is triggered by the tower current, whereas the latter records the peak energies of events and is working continuously. The Uppsala scintillator, whose data are presented in this report, is composed of a cylindrical NaI(Tl) crystal of 76 mm thickness and 76 mm diameter, with an effective energy range of $\sim$ 20 keV to 2 MeV, a temporal resolution of $\sim$1/4 $\upmu$s, and is of the same design as that used by Hettiarachchi et al.; refer to their 2018 paper^9^ for a detailed description. This X-ray detector is connected to the same digitiser as our Radome E-field probe and therefore by default synchronised; synchronisation of these two with the tower current and current derivative signals (which are themselves synchronised in the same manner) is done by aligning the time of the first E-field “step” with the time of the $\dot{B}$ extremum associated with the first current pulse, as these tend to be the sharpest. The HSC and “far” E-field data are synchronised with the rest by GPS time-stamp if the antennae are functional at the time of the flash. If not, manual synchronisation can be carried out via waveform matching. More detailed information on the Säntis measurement system can be found in Rachidi and Rubinstein^10^.

All computational data analysis and presentation were carried out using the Python programming language, with the NumPy, SciPy and Matplotlib libraries in particular.

## Results

**Table 2:** Lightning flashes analysed—data summary.

Flash	Date UTC	Type	Prior activity	Current (& derivative)	High-speed camera	Interfer-ometer	E-field (20-m & 15-km)
UP0	2021-06-28 23:26:53	2	Yes	Yes	No	No	Yes
UP1	2021-07-24 16:06:07	2	No	Yes	No	Yes	Yes
UP2	2021-07-24 16:24:03	1	No	Yes	Yes	No	Yes
UP3	2021-07-30 18:00:10	2	Yes	Yes	No	Yes	Yes

We analysed 4 upward positive and 8 upward negative flashes with associated X-ray emissions that occurred during the Summer 2021 thunderstorm season [A comprehensive analysis of all 12 flashes will be the subject of a separate paper]. The data available for the 4 upward positive flashes (UPFs) presented here are summarised in Table 2 [Only near E-field waveforms are presented and analysed herein]. Romero et al.^11^ classified UPFs into two categories: Type 1 flashes, which exhibit a large unipolar return stroke–like current pulse following the upward negative stepped-leader phase; and Type 2 flashes, which do not feature such a large pulse and consist solely of a 100 millisecond-scale waveform with large, oscillatory pulse trains due to upward negative stepped leaders.

In addition to tower current and electric field measurements, two (UP1 and UP3) were recorded by the interferometer (subject to a separate analysis), and one (UP2) was captured by the high-speed camera. UP0 and UP3 also saw preceding lightning activity in the vicinity, as identified from their E-field waveforms, occurring within $\sim$150 ms prior to the onset of the stepped leader. Upward lightning flashes from tall structures have been classified into two categories: (i) self-initiated and (ii) other-triggered (Wang et al.^13^). This classification is based on whether there is lightning activity in the geographical and temporal vicinity of the tower-initiated flash. A tower flash falls into the self-triggered category if it is not preceded by any lightning (cloud-to-ground or cloud) within a predefined circular area around the tower and within a specific time interval prior to the tower flash. Conversely, flashes categorised as other-triggered are preceded by cloud-to-ground or cloud flash activity within a predefined distance of the tower and within a prior time interval constraint with respect to the tower flash. Observations and theoretical analyses^14,15^ have suggested that nearby lightning activity could trigger upward lightning.

It should be noted that flashes UP1, UP2, and UP3 occurred during the Laser Lightning Rod project presented in Houard et al.^16^ (therein called L1, L2, and L3, respectively), while the laser was on, whereas flash UP0 did not. The presence of the laser beam does not have an obvious effect on X-ray production: firstly, though the number of X-ray events per flash was found to be higher in the presence of the laser beam, the number of observed flashes was too small to draw definitive conclusions; secondly, laser-guided lightning was observed over a distance of about 50 m (their Fig. 2), but with no evidence of laser-induced lightning *initiation*^16^.

Here, we define the initial continuous current (ICC) at the start of the leader, i.e., the first significant deviation from zero of the electric field, current and current-derivative. For the latter two, we have chosen the convention of a negative current corresponding to a positive charge transfer from cloud to ground. Each positive flash had between one and seven distinct X-ray events associated with this “stepping” of the upward negative leader, also indicated by pulses in the current waveform.

**Table 3:** UP flash leader steps with associated X-rays.

Flash	$t_{SL}$ [$\mu$s]	$I_p$ [kA]	$t_{mr}$ [$\mu$s]	|$\frac{dI}{dt}$| [$\frac{\textrm{kA}}{\mu \textrm{s}}$]	$\Delta E$ [$\frac{\textrm{V}}{\textrm{m}}$]	$t_{Er}$ [$\mu$s]	XRE [keV]
UP0	150.9	0.81±0.05	0.13$^{+0.02}_{-0.01}$	6.2±0.4	545±55	0.15	63.4±0.9
UP1	0.0	1.66±0.05	0.12$^{+0.01}_{-0.01}$	14.2±0.3	1025±40	0.20	256.1±0.7
	89.5	2.01	0.24$^{+0.02}_{-0.02}$	8.2	885	0.30	85.1
	334.6	1.91	0.88$^{+0.18}_{-0.14}$	2.2	635	9.50	87.0
	345.7	2.89	0.79$^{+0.09}_{-0.08}$	3.7	635	9.50	55.0
	465.6	2.20	1.53$^{+0.50}_{-0.32}$	1.4	330	10.55	31.6
	554.1	2.80	1.36$^{+0.29}_{-0.21}$	2.1	590	17.10	79.8
	774.0	2.66	1.78$^{+0.55}_{-0.35}$	1.5	350	19.50	31.1
UP2	46.3	1.14±0.05	0.13$^{+0.01}_{-0.01}$	8.7±0.3	1450±40	0.20	54.6±0.7
	267.5	0.41	0.31$^{+0.14}_{-0.09}$	1.3	280	11.05	44.1
UP3	118.0	2.44±0.05	0.47$^{+0.04}_{-0.04}$	5.2±0.3	720±40	0.25	26.5±0.8
	355.0	2.71	1.28$^{+0.26}_{-0.19}$	2.1	475	2.35	50.4
	483.0	1.82	1.08$^{+0.30}_{-0.20}$	1.7	175	6.70	53.0
	785.0	5.20	2.52$^{+0.51}_{-0.37}$	2.1	565	21.10	42.8
$\mu _a \pm \sigma _a$	–	2.19±1.11	0.90±0.71	4.3±3.7	620±320	7.75±7.30	68.6±55.2
${\mu _g}^{\mu _g(\sigma _g - 1)}_{\mu _g(1-1/\sigma _g)}$	–	$1.88^{+1.55}_{-0.85}$	$0.59^{+1.05}_{-0.38}$	$3.2^{+3.6}_{-1.7}$	$540^{+375}_{-220}$	$2.60^{+14.90}_{-2.20}$	$57.0^{+41.5}_{-24.0}$

Table 3 presents the measured data for each leader step with correlated X-rays. The time $t_{SL}$ (“stepped leader”) is measured from the onset of the ICC. $I_p$ represents the absolute value peak current of a given pulse, and |*dI*/*dt*|$_{\textrm{max}} \,$ its maximum current derivative (slope). Together they make the minimum current rise-time, defined by Giri et al.^17^ as:

1 $$\begin{aligned} t_{mr} = \frac{I_p}{\Vert \frac{dI}{dt}\Vert _{\textrm{max}}} \end{aligned}$$

which has a temporal accuracy of 20 ns. The change in the electric field is given by $\Delta E$ and its 80% rise-time by $t_{Er}$ (with a temporal resolution of 50 ns). Finally, XRE is the associated X-ray energy. The first row of each flash provides the error associated with sensor noise at that time, with the exception of the calculated $t_{mr}$, whose errors vary with measurement. The last two rows of the table provide the arithmetic and geometric means ($\mu _a$, $\mu _g$) and standard deviations ($\sigma _a$, $\sigma _g$) of each data set above. One can already see that all parameters except $I_p$ exhibit at least some degree of temporal variation, as will be confirmed later in "Discussion" Section.

**Figure 2:**
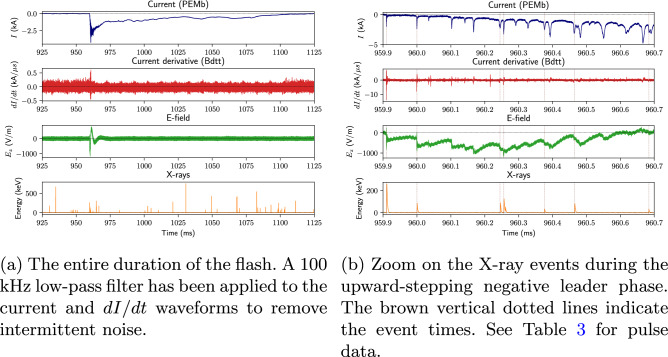
Waveforms of UP1, a Type 2 upward positive flash that occurred on July 24, 2021 at 16:06:07 UTC. “PEMb” and “Bdtt” specify the bottom Rogowski coil and top $\dot{B}$ sensor, respectively. $E_z$ is the measured vertical component of the electric field at 20 m. The time is from the beginning of the recording ($\sim$1 s before the current peak).

UP1, whose current, *dI*/*dt*, near E-field, and X-ray waveforms of are shown in Fig. 2, lacks the aforementioned “main” pulse, and is therefore categorised as a Type 2 upward positive flash (Fig. 2a). Note that there are many background X-ray flux events (likely cosmic ray secondaries) that are not undeniably associated with the flash.[Given our sensor’s detection rate of $\sim$110 background events per second, the probability of one of these occurring during the $\sim$1.5 $\upmu$s duration of a leader step is < 0.02%]. Figure 2b presents an expanded view of the initiation of the upward leader and its stepping; the electric field steps are clearly shown to be associated with ICC pulses, 1/3 of which were associated with X-ray emissions. As X-rays are considered to be emitted omnidirectionally in the case of upward stepped leaders^18,19^, the absence of detection for all leader steps is likely due to limited sensor area and dynamic range. The 7 X-ray pulses had a median temporal separation on the order of 100 $\upmu$s, and median energy on the order of 80 keV. Note, however, the decrease in pulse peak energy as time goes on.

**Figure 3:**
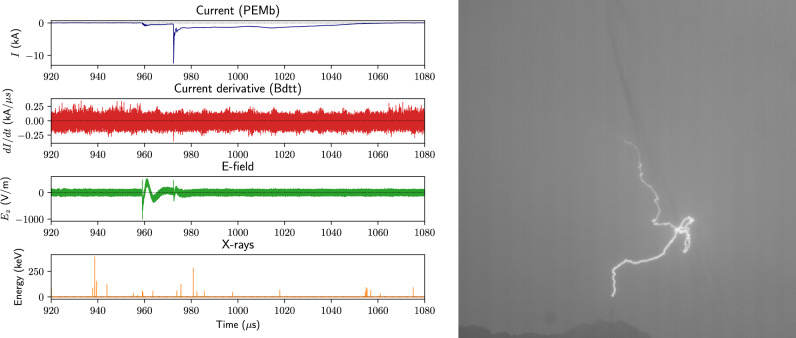
Waveforms and integrated HSC frames of UP2, a Type 1 upward positive flash that occurred on July 24, 2021 at 16:24:03 UTC. A 100 kHz low-pass filter has been applied to the current and *dI*/*dt* waveforms to remove intermittent noise. See Fig. 4 for a zoom-in view.

**Figure 4:**
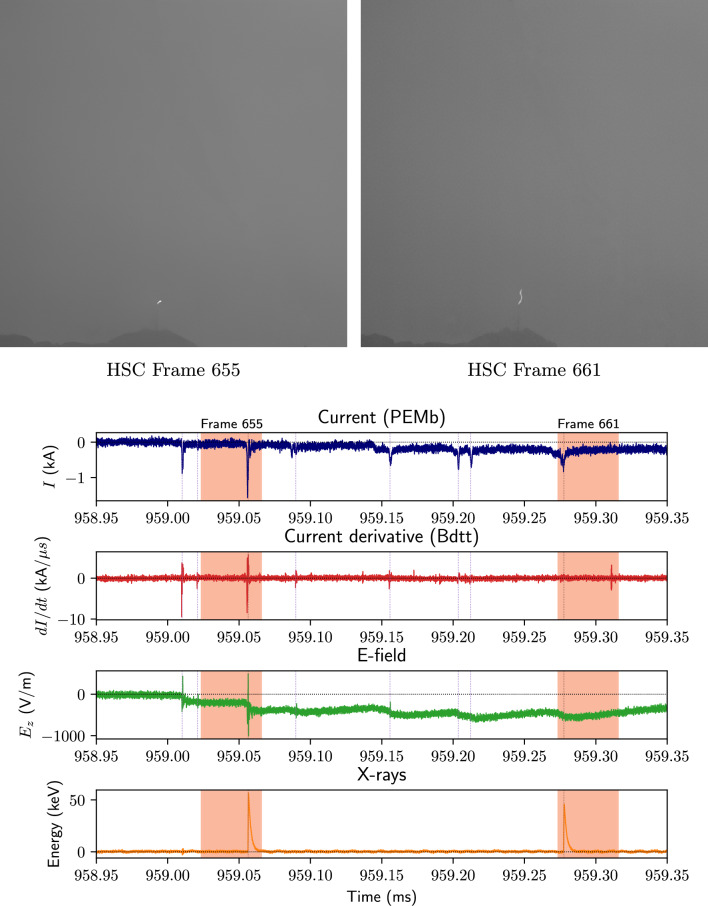
X-ray events during the upward stepping negative leader phase of UP2. HSC frames containing the two X-ray pulses observed are shown above, and their approximate temporal width ($\sim$42 $\upmu$s) is highlighted by the red-shaded regions in the waveforms below. E-field steps without associated X-rays are also indicated by the vertical dotted violet lines. See Table 3 for pulse data and Fig. 3 for a zoomed-out view of the waveforms.

The whole flash waveforms and integrated HSC frames of UP2 are shown in Fig. 3. This was clearly a Type 1 upward positive flash^11^, with a very obvious return stroke–like pulse after the upward-stepping leader. In the right-hand panel of the figure, one can make out the Säntis tower, from which the flash initiated, at the base of the rather tortuous plasma channel [The faint black streak running diagonally across the integrated stills are raindrops streaming down the camera’s protective window pane]. The plots at the bottom of Fig. 4 provide a zoom on the beginning of the ICC, when the two X-ray pulses occurred and the top pictures are HSC stills containing these pulses, which occurred 221 $\upmu$s apart with an average energy of 49 keV. Once again, these are clearly associated with the leader-stepping process, though unlike UP1, only 1/10 of the leader steps had accompanying X-rays detected. This may be due to the lower current amplitudes observed during the leader-propagation phase of UP2 ($\sim$ 0.8 kA) when compared to UP1 ($\sim$2.4 kA).

UP3 was a Type 2 flash like UP1; its 4 X-ray pulses (about 1/6 of all ICC pulses) had a mean temporal separation of $\sim$220 $\upmu$s and an average energy of 43 keV. UP0 was likely also a Type 2 flash (though with two relatively large M-component—like ICC pulses); its singular X-ray pulse (< 5% of all ICC pulses) had an energy of 63 keV. These flashes’ waveforms are not depicted here for the sake of conciseness, though all their leader step data have been included in the following analysis. See Supplementary information for plots.

## Discussion

**Figure 5:**
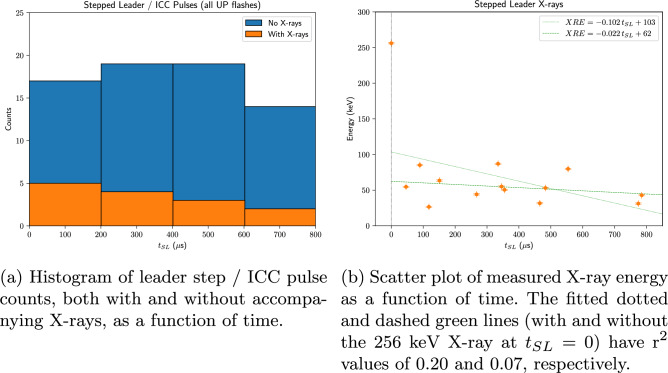
Plots depicting the temporal dependence of the X-ray counts and pulse energy for flashes UP0, UP1, UP2 and UP3. Time $t_{SL} = 0$ is set to the start of the stepped leader/ICC. Data taken from Table 3. r$^2$ is the coefficient of determination.

Figure 5 shows how both measured X-ray count and energy decrease as functions of time from the onset of the stepped leader, $t_{SL}$. Note how leader steps / ICC pulses with measured accompanying X-rays compose a steadily decreasing percentage of all measured pulses, starting at $\sim$ 29% during the first 200 $\mu$s, and dropping to 0% after 800 $\upmu$s. Although one can readily argue that this count decrease observed in Fig. 5a is simply due to the diminishing photon flux at the sensor location as the leader tip (where the X-rays are presumed to be emitted^20,21^) moves away, the same argument cannot be made for *energy* decrease plotted in Fig. 5b, as the waveforms are indicative of single events, rather than photon-burst energy pile-ups (compare with Fig. 4 of Saleh et al.^5^). The best-fit lines in this plot (affine fits both with and without the 256 keV X-ray at $t_{SL} = 0$, for reasons discussed below) imply the existence of a finite time (>1 ms) after which X-rays would no longer be detected. This is consistent with the expectation of X-rays losing energy to the intervening air via Compton scattering.

**Figure 6:**
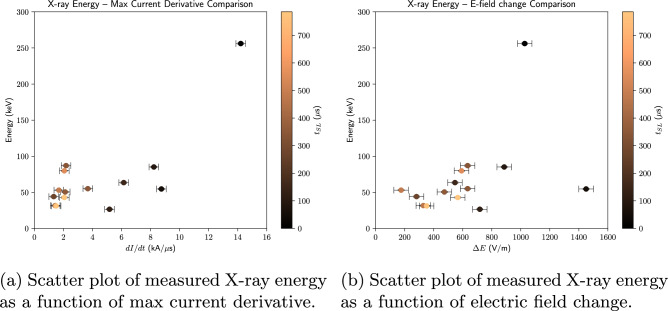
Color-mapped scatter plots depicting the parametric dependence of the X-ray energy for flashes UP0, UP1, UP2 and UP3, similar to Figure 15 of Mallick et al.^6^. Time $t_{SL} = 0$ is set to the start of the stepped leader / ICC. Data taken from Table 3.

Figure 6a shows a scatter plot of measured X-ray energy versus maximum current derivative, |$\frac{dI}{dt}$|$_{\textrm{max}}$. It is clear from the color map that the latter also decreases as a function of time $t_{SL}$ (alternatively, the current pulse rise-time $t_{mr}$ increases). One can reasonably fit an exponential curve, i.e., $XRE \propto \exp (\frac{dI}{dt})$, with an r$^2$ value of 0.79 to this dataset. It should be noted, however, that if one refits the data excluding the 256 keV X-ray at $t_{SL} = 0$, this trend vanishes (negative r$^2$ value). One motivation for doing so is that, besides being $>2\sigma$ above both $\mu _a$ and $\mu _g$, this particularly energetic photon was the only one associated with the first leader step, which initiates from the tip of the tower rather than an extending plasma channel, and therefore originates under different physical parameters.

Figure 6b shows a scatter plot of X-ray energy versus electric field change, $\Delta E$. It is clear from the color map that $\Delta E$ decreases as a function of time, as does the X-ray energy, albeit to a lesser extent (see Fig. 5b). The former can be at least partly explained by the fact that our sensor measures only the vertical component of the electric field, and the leader tip is moving away from the tower. Furthermore, the X-ray energy appears to increase with $\Delta E$ (r$^2 = 0.18$ for an affine fit). As before, if we refit without the highest energy event, the already-weak trend becomes even more tenuous (r$^2 = 0.09$).

A relationship between the presence of X-ray emissions and high parameter values does remain consistent, however. Tables 4 and 5 present a comparison of the average current derivative maxima and electric field changes (both arithmetic and geometric means) for leader steps with and without accompanying X-rays. By both metrics, steps accompanied by X-rays have markedly larger current derivative maxima and E-field changes. The ratios of the arithmetic means with and without X-rays for all flashes analysed are about 1.6 for both current derivative maxima and electric field changes.

**Table 4:** Average current derivative maxima ($\mu _a \pm \sigma _a$ |  ${\mu _g}^{\mu _g(\sigma _g - 1)}_{\mu _g(1-1/\sigma _g)}$ kA/$\mu$s).

	With X-rays	Without X-rays	All
UP0	6.2 (one event)	2.0±1.3 | $1.7^{+1.1}_{-0.7}$	2.4±1.7 | $2.0^{+1.6}_{-0.9}$
UP1	4.8±4.4 | $3.3^{+4.2}_{-1.8}$	2.5±1.1 | $2.4^{+1.0}_{-0.7}$	3.3±2.9 | $2.6^{+2.1}_{-1.2}$
UP2	5.0±3.7 | $3.4^{+5.3}_{-2.1}$	2.7±2.4 | $2.2^{+1.6}_{-0.9}$	3.1±2.8 | $2.4^{+2.1}_{-1.1}$
UP3	2.8±1.4 | $2.5^{+1.3}_{-0.9}$	3.1±2.2 | $2.6^{+1.9}_{-1.1}$	3.0±2.1 | $2.6^{+1.7}_{-1.0}$
All	4.3±3.7 | $3.2^{+3.6}_{-1.7}$	2.7±1.9 | $2.3^{+1.5}_{-0.9}$	3.0±2.5 | $2.5^{+1.9}_{-1.1}$

**Table 5:** Average electric field changes ($\mu _a \pm \sigma _a$ |  ${\mu _g}^{\mu _g(\sigma _g - 1)}_{\mu _g(1-1/\sigma _g)}$ V/m).

	With X-rays	Without X-rays	All
UP0	545 (one event)	300±75 | $290^{+70}_{-55}$	320±100 | $310^{+95}_{-75}$
UP1	635±235 | $590^{+285}_{-195}$	360±110 | $345^{+110}_{-85}$	455±210 | $415^{+210}_{-140}$
UP2	865±585 | $640^{+810}_{-355}$	380±155 | $360^{+120}_{-90}$	455±320 | $390^{+230}_{-145}$
UP3	485±200 | $430^{+305}_{-180}$	420±300 | $355^{+245}_{-145}$	430±285 | $370^{+260}_{-150}$
All	620±320 | $540^{+375}_{-220}$	375±205 | $340^{+160}_{-110}$	425±255 | $375^{+220}_{-140}$

It has been suggested^22–24^ that the so-called “cold runaway electron mechanism”, as opposed to the relativistic runaway electron avalanche (RREA) model proposed by Gurevich et al.^25^, is active in X-ray emissions associated with lightning leaders. For the cold runaway mechanism to be active, it is necessary for the background electric field at atmospheric pressure to exceed about 20 MV/m^21,23^. If the electric field increases slowly in atmospheric air, as its value reaches around 3 MV/m the normal electrical breakdown process takes over and the resulting increase in conductivity of the discharge channel limits further increase of the electric field strength. The electric field will therefore be clamped to a value equal to or below this breakdown threshold.[If the pressure is below atmospheric, these mechanisms remain the same except that the threshold fields are scaled down linearly with pressure.] Since a certain amount of time is needed for the completion of standard breakdown, in order to achieve the cold runaway mechanism the electric field has to increase very rapidly in a given region of space so that there isn’t sufficient time for the standard breakdown mechanism to take over and clamp the electric field at $\sim$3 MV/m. Thus, only very fast discharge processes (sub-microsecond scale) can generate the strong electric fields needed to push electrons into the cold runaway regime quickly enough^23^. This is in agreement with the observation that X-ray emissions occur during discharge processes with rapidly changing currents (the leader steps), such as those seen in this study. Furthermore, the X-rays we observed in synchronization with the leader-stepping process had energies on the order of 50 keV, as opposed to the MeV-range energies ($\gamma$-rays) associated with the RREA model, a.k.a. “*hot* runaway electron mechanism”^18^.

## Conclusion

Herein we reported, to the best of our knowledge, the first measurements of X-rays produced by positive lightning flashes, specifically during the stepping of the upward negative leader. We presented the waveforms of the current, current derivative, electric field, and X-ray energy for the four upward positive flashes in question (one Type 1 and three Type 2), as well as high-speed camera stills for one of them (the Type 1 upward positive flash). These time-synchronised data served to confirm that the X-ray emissions detected are associated with the initial steps of the upward negative leader. Further analysis of the parameters at play revealed four additional points of interest:

- The frequency and energy of X-ray pulses appear to decrease as functions of time, with pulses disappearing altogether within the first millisecond of leader initiation, consistent with a receding source and increased Compton scattering;
- Lower current amplitudes during the leader-stepping phase appear to be correlated with lower percentages of steps with accompanying X-rays, which also end sooner;
- Leader steps accompanied by X-rays have markedly larger current derivative maxima and E-field changes compared to leader steps not accompanied by X-rays;
- This association of X-ray emission with the maximum current-derivative and electric field change of leader steps, as well as the relatively low photon energies detected, support the cold runaway electron model as the active mechanism for lightning leader X-ray production.

These observations contribute to improving our understanding of upward lightning, and will soon be followed by a review including X-ray–emitting upward *negative* flashes observed at the Säntis tower, and simultaneous interferometric data gathered during the summer 2021 experimental campaign.

## Supplementary Information

Supplementary Figures.

## Data Availability

All processed data analysed during this study are included in this published article (and its supplementary information files). Raw data sets generated during the current study are available from the corresponding author on reasonable request.

## Code availability

Upon request from corresponding author.
