# Supplementary material for: Direct observations of X-rays produced by upward positive lightning
Source: Sci Rep. 2024 Apr 6;14:8083. doi: 10.1038/s41598-024-58520-x (PMC11372170; doi:10.1038/s41598-024-58520-x)
Supplement: Supplementary file 1 — Supplementary Figures. [file 41598_2024_58520_MOESM1_ESM.pdf]

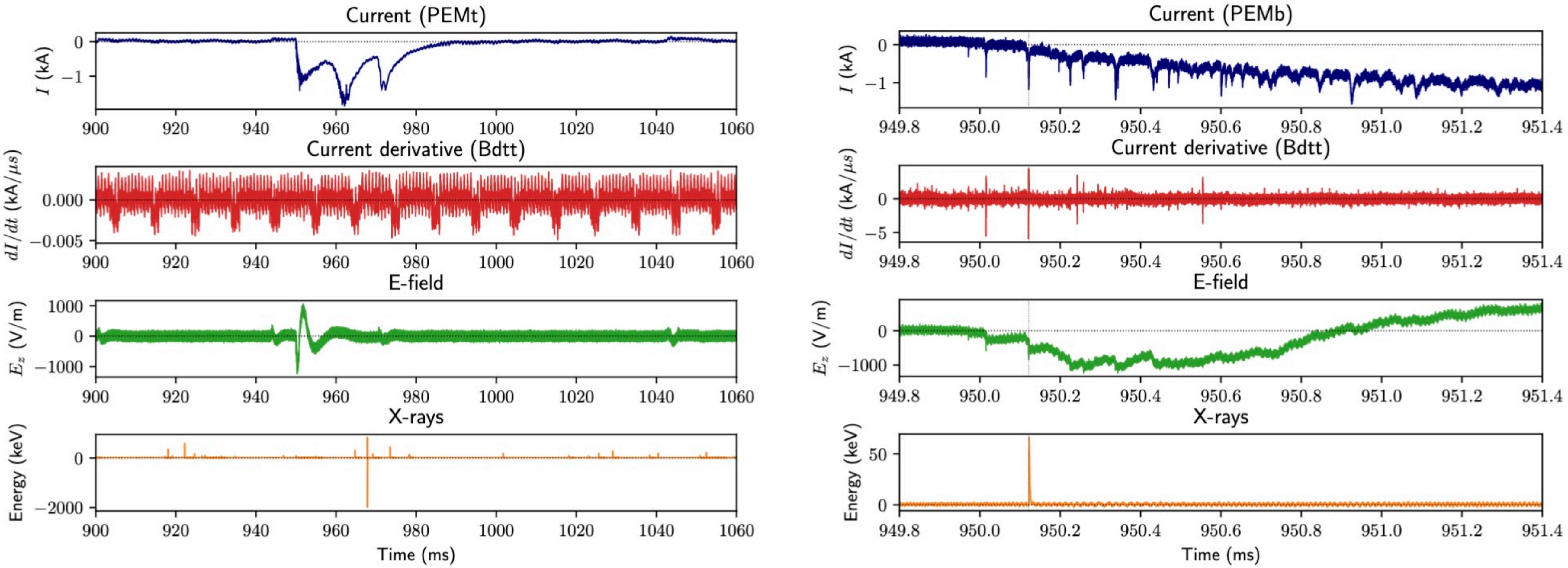

(a) The entire duration of the flash. A 100 kHz low-pass filter has been applied to the current and dI/dt waveforms to remove intermittent noise. The large negative overshoot in X-ray energy is due to detector saturation.

(b) Zoom on the X-ray event during the upward stepping negative leader phase. The brown vertical dotted lines indicate the event times. See Table 3 for pulse data.

Fig. A1: Data associated with the Type 2 upward positive flash UP0, that occurred on June 28, 2021 at 23:26:29 UTC. "PEMb" and "Bdtt" specify the bottom Rogowski coil and top $\dot{B}$ sensor, respectively. E_z is the measured vertical component of the electric field. The time is from the beginning of the recording (~1 second before the current peak).

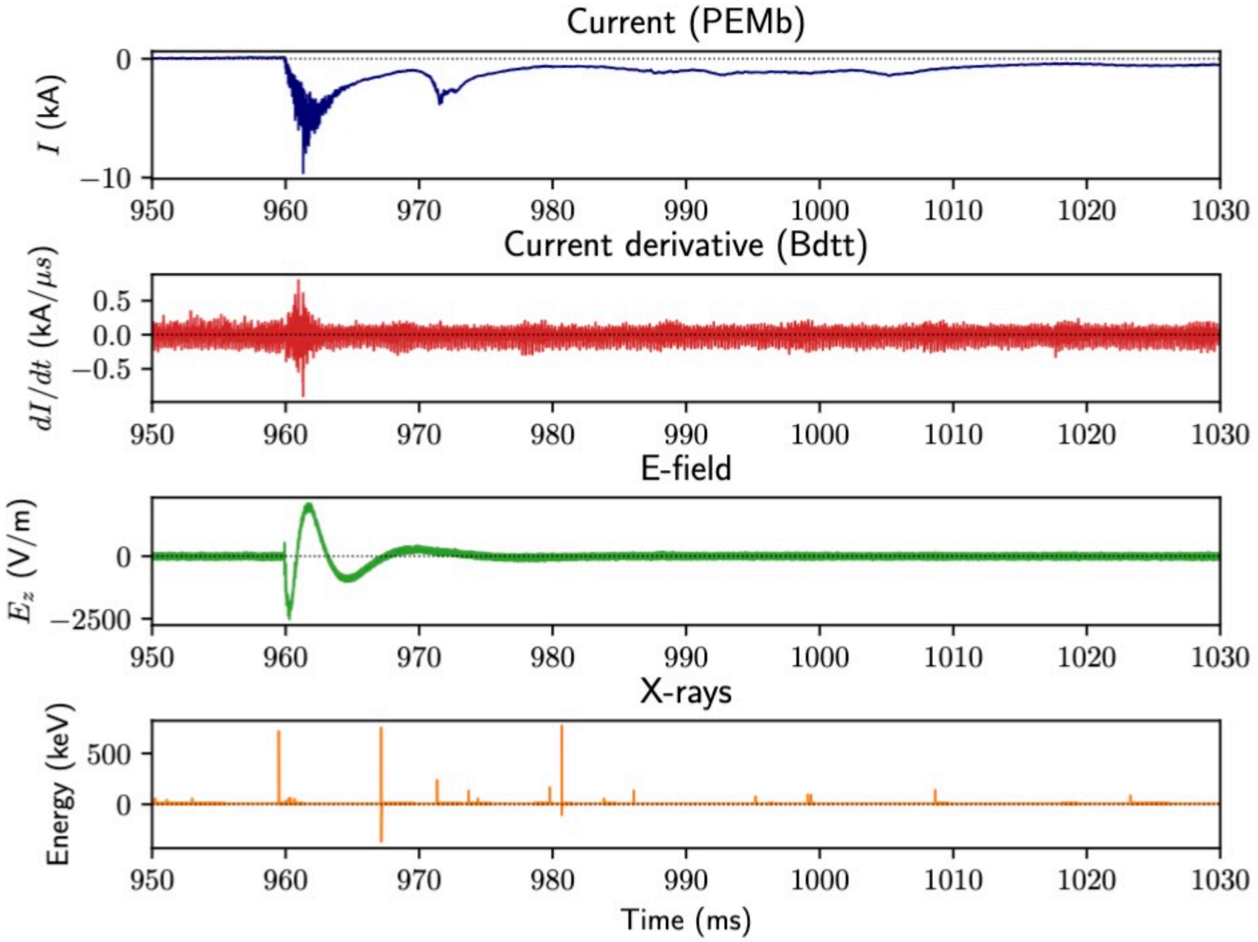

(a) The entire duration of the flash. A 100 kHz low-pass filter has been applied to the current and dI/dt waveforms to remove intermittent noise. Negative X-ray energies are due to detector saturation.

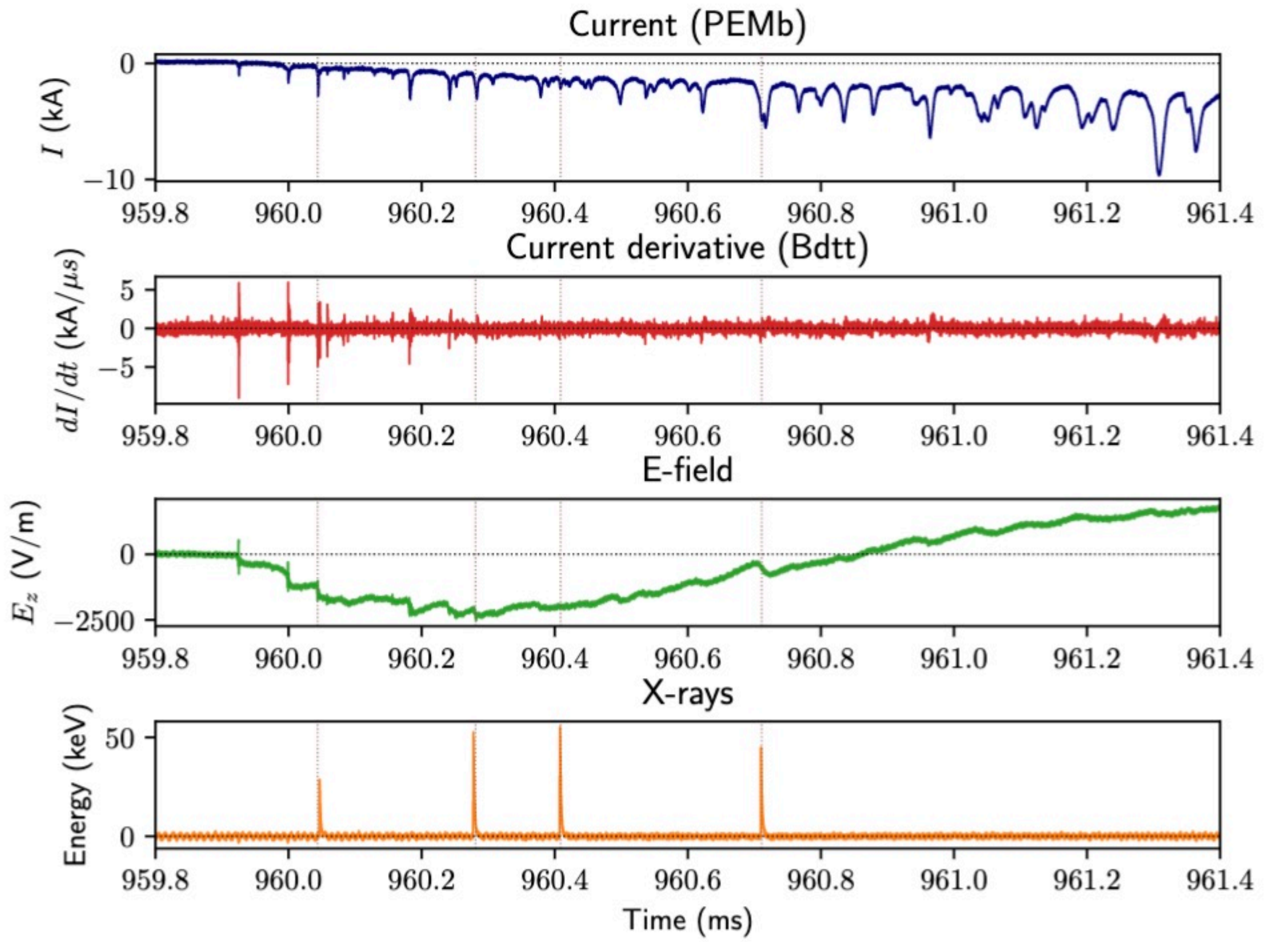

(b) Zoom on the X-ray events during the upward stepping negative leader phase. The brown vertical dotted lines indicate the event times. See Table 3 for pulse data.

Fig. A2: Data associated with the Type 2 upward positive flash UP3, that occurred on July 30, 2021 at 18:00:10 UTC. "PEMb" and "Bdtt" specify the bottom Rogowski coil and top $\dot{B}$ sensor, respectively. E_z is the measured vertical component of the electric field. The time is from the beginning of the recording (~1 second before the current peak).

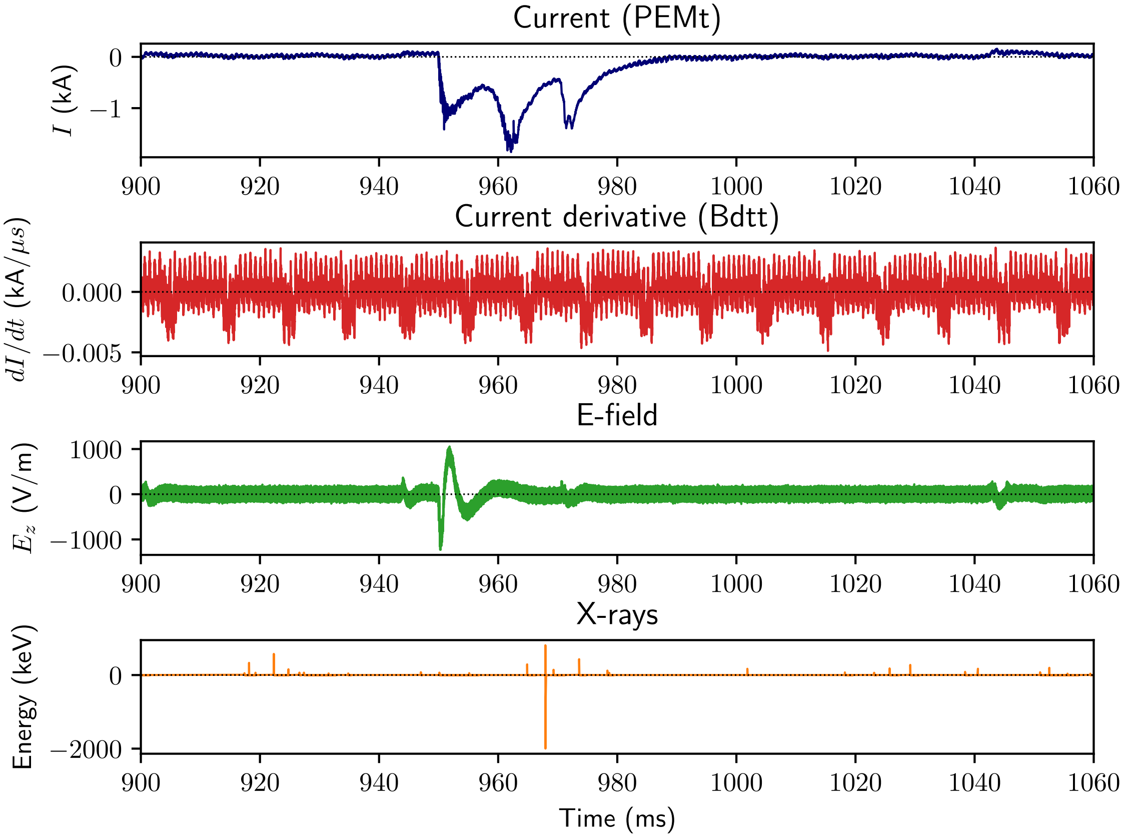

Current (PEMt)
I (kA)
0
−1
Current derivative (Bdtt)
dI/dt (kA/μs)
0.000
−0.005
E-field
E_z (V/m)
1000
0
−1000
X-rays
Energy (keV)
0
−2000
900
920
940
960
980
1000
1020
1040
1060
Time (ms)

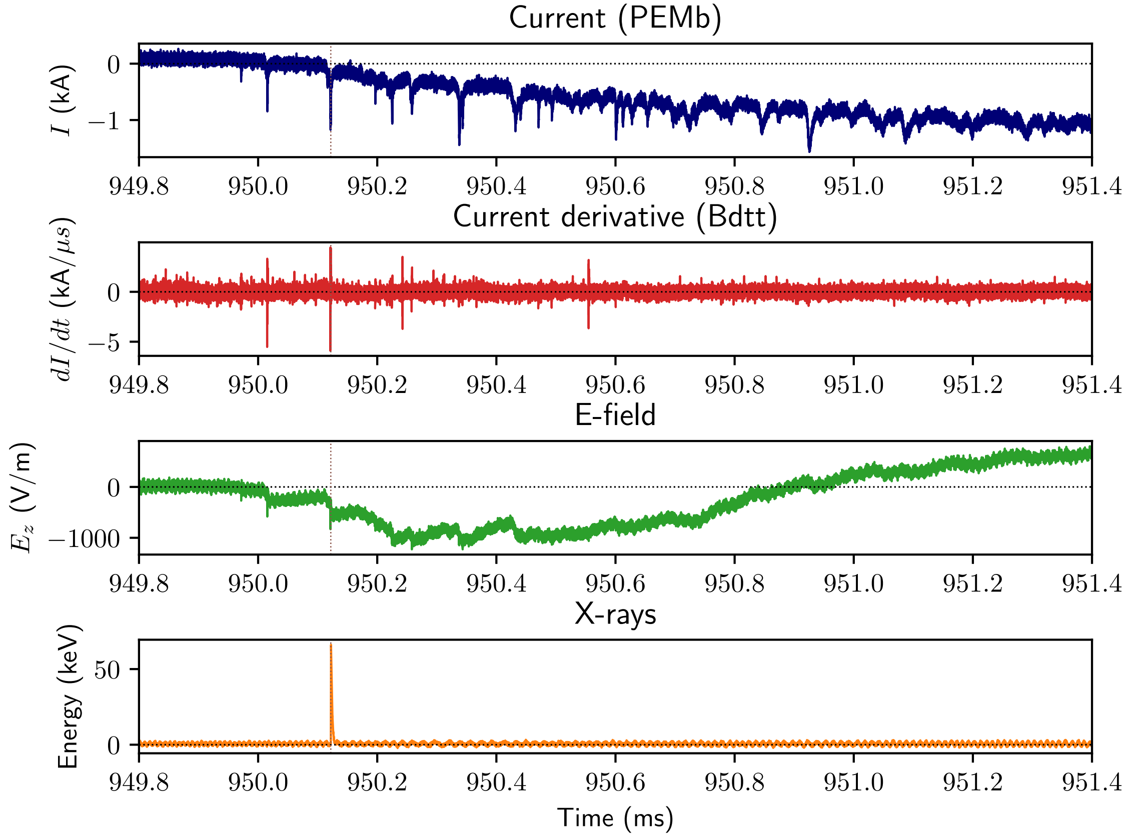

Current (PEMb)
I (kA)
0
−1
Current derivative (Bdtt)
dI/dt (kA/μs)
0
−5
E-field
E_z (V/m)
0
−1000
X-rays
Energy (keV)
50
0
949.8
950.0
950.2
950.4
950.6
950.8
951.0
951.2
951.4
Time (ms)

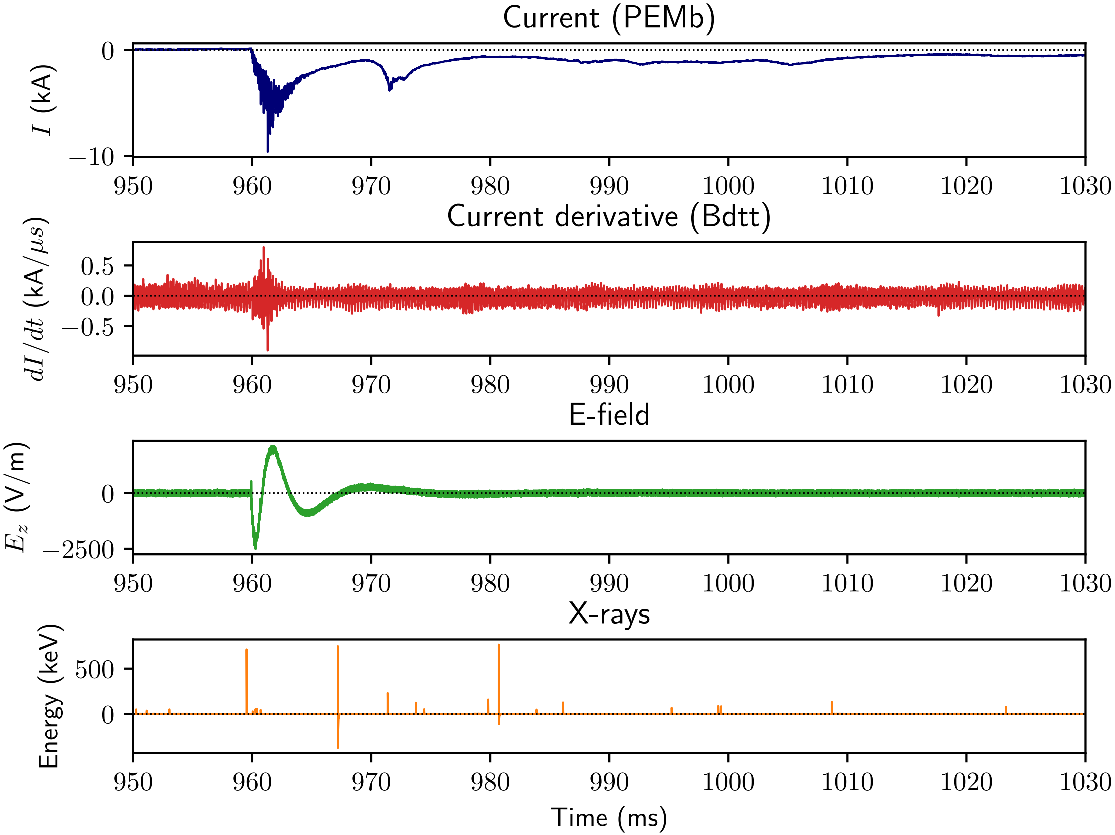
Current (PEMb)
I (kA)
0
-10
950
960
970
980
990
1000
1010
1020
1030
Current derivative (Bdtt)
dI/dt (kA/μs)
0.5
0.0
-0.5
E-field
E_z (V/m)
0
-2500
X-rays
Energy (keV)
500
0
Time (ms)

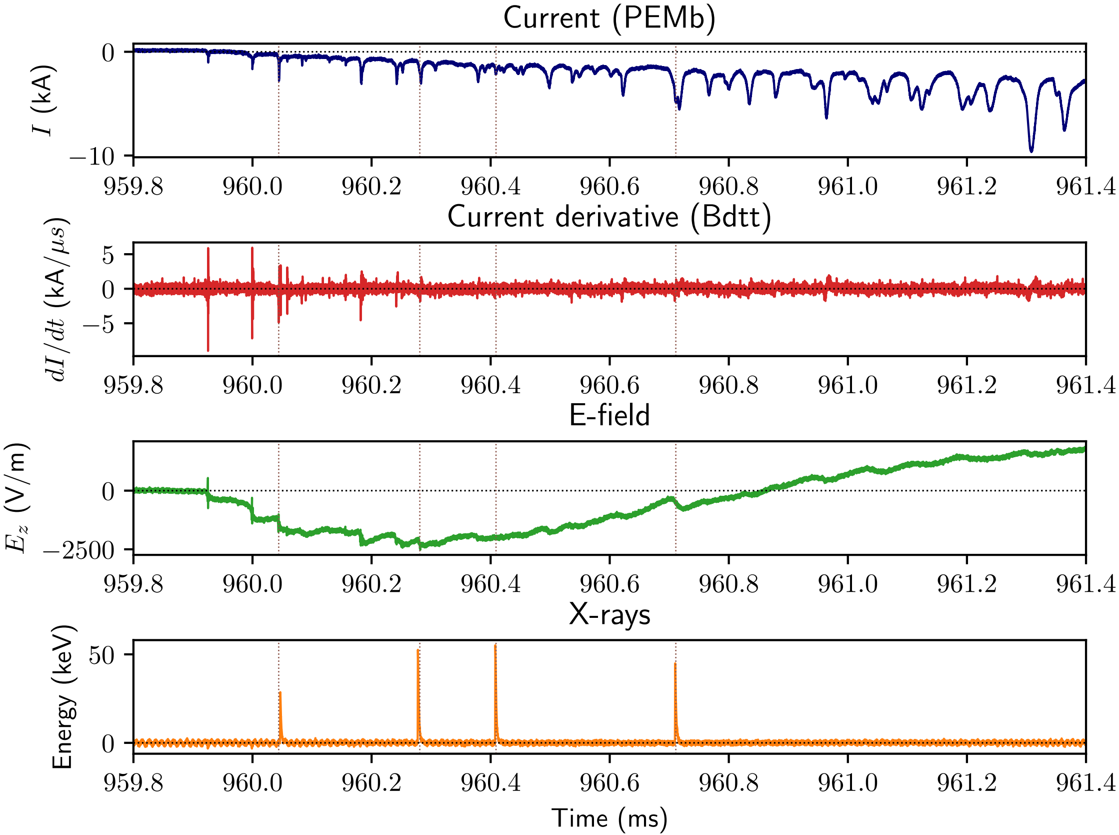
Current (PEMb)
I (kA)
0
−10
Current derivative (Bdtt)
dI/dt (kA/μs)
5
0
−5
E-field
E_z (V/m)
0
−2500
X-rays
Energy (keV)
50
0
959.8
960.0
960.2
960.4
960.6
960.8
961.0
961.2
961.4
Time (ms)
